# Different Combinations of Behavior Change Interventions and Frequencies of Interpersonal Contacts Are Associated with Infant and Young Child Feeding Practices in Bangladesh, Ethiopia, and Vietnam

**DOI:** 10.1093/cdn/nzz140

**Published:** 2019-12-09

**Authors:** Sunny S Kim, Phuong Hong Nguyen, Lan Mai Tran, Silvia Alayon, Purnima Menon, Edward A Frongillo

**Affiliations:** 1 Poverty, Health, and Nutrition Division, International Food Policy Research Institute, Washington, DC, USA; 2 Save the Children, Washington, DC, USA; 3 University of South Carolina, Columbia, SC, USA

**Keywords:** coverage, counseling, infant and young child feeding, interpersonal communication, Bangladesh, Ethiopia, Vietnam

## Abstract

**Background:**

Social and behavior change communication interventions are integral to improving dietary and care practices, but evidence on the impact of the combination and intensity of these interventions in different contexts is scarce.

**Objectives:**

We examined the extent of and factors associated with intervention exposure: interpersonal communication (IPC) alone or with other interventions (i.e., mass media, community mobilization, or nutrition-sensitive agricultural activities), number of and factors associated with IPC contacts, and combinations of intervention components and number of contacts associated with infant and young child feeding (IYCF) practices.

**Methods:**

We used endline survey data from impact evaluations in Bangladesh, Ethiopia, and Vietnam (*n* = 1001, 1720, and 1001 mothers with children aged <2 y, respectively). Multivariable regression models were used for analyses.

**Results:**

Exposure to the interventions varied in all 3 countries. On average, mothers received 8 visits in the last 6 mo in Bangladesh, 2 visits in the last 3 mo in Ethiopia, and 1 visit in the last 6 mo in Vietnam. Across countries, the factors associated with intervention exposure and number of IPC contacts differed. In Ethiopia, exposure to IPC with other interventions was associated with higher odds of achieving minimum meal frequency (OR: 1.6), minimum dietary diversity (OR: 1.8), and consumption of iron-rich foods (OR: 4.7). In Vietnam, exposure to IPC alone or with mass media was associated with higher odds of exclusive breastfeeding (EBF; OR: 2.8–3.7). Near-monthly visits were associated with 2–3 times higher odds of IYCF practices in Bangladesh and Ethiopia. In Vietnam, even 1 IPC visit was associated with 2 times higher odds of EBF.

**Conclusions:**

Exposure matters for impact, but the combination of behavior change interventions and number of IPC contacts required to support IYCF behavior change are context specific. This trial was registered at www.clinicaltrials.gov as NCT01678716 (Bangladesh), NCT02775552 (Ethiopia), and NCT01676623 (Vietnam).

## Introduction

Social and behavior change communication (SBCC) is an integral component of nutrition programs aiming to improve dietary and care practices. Interpersonal communication or counseling has been shown to be an effective behavior change intervention for improving breastfeeding and complementary feeding practices (1–7). The design of these interventions in terms of content, intensity, mode of communication, delivery platform, etc., varies extensively from study to study.

To guide the design, implementation, and scaling up of programs for breastfeeding counseling, the WHO recently developed a guideline on global, evidence-informed recommendations on breastfeeding counseling (8). The guideline recommends that breastfeeding counseling should be provided in the antenatal and postnatal periods and up to 24 mo or longer, through face-to-face or interpersonal counseling and additionally via remote modes, and should be provided ≥6 times throughout this period. These recommendations and others were based on low- to moderate-quality evidence across 63 studies (8). There is little specificity in the timing and frequency or intensity of counseling, however, and the guideline highlights further research needs for studies across different regions, countries and population groups, and contexts (e.g., in areas where breastfeeding is the norm and where breastfeeding practices are not optimal). Questions also remain about coverage, who are exposed, and what combination and intensity of behavior change interventions are feasible and needed by caregivers to have impact on their child feeding behaviors, and/or if these combinations and intensities differ by context.

Evidence on the impact of counseling intensity is mixed, but many studies suggest that more intensive programs and counseling (e.g., number of sessions) (9–12) and individual (vs. group) sessions (10, 12) may be more effective. A recent Cochrane review of community-based maternal and newborn educational care packages showed that integrating interventions with educational components delivered by community workers in group settings was associated with positive newborn health-related behaviors and outcomes (13) despite large heterogeneity of programs in terms of the promoted behaviors, delivery context, and characteristics of the target population.

Alive & Thrive is an initiative that aims to improve infant and young child feeding (IYCF) practices through large-scale SBCC programs, which include interpersonal communication (IPC) delivered by frontline health workers (14). IPC during health facility or home visits was combined with mass media (MM) and community mobilization (CM) activities, which were delivered by a nongovernmental organization in Bangladesh and government health systems in Ethiopia and Vietnam. Over a 4-y period (2010–2014), program interventions led to large significant impacts on IYCF practices in both Bangladesh (15, 16) and Vietnam (17); in Ethiopia, impacts on complementary feeding practices and stunting were observed after a 2-y period (2015–2017) (18).

In this article, we examined the coverage and frequency of IPC with or without other SBCC interventions [MM, CM, and nutrition-sensitive agricultural (AG) activities in Ethiopia] across the 3 diverse country settings. Our objectives were to understand the following: *1*) the extent of exposure to the large-scale behavior change interventions in the 3 countries and the household, maternal, and child factors associated with intervention exposure; *2*) the number of IPC contacts achieved and the factors associated with the number of contacts; and *3*) the association between different combinations of interventions and number of contacts and IYCF practice outcomes.

## Methods

### Study context and intervention description

This study used data from the impact evaluation of Alive & Thrive's intervention programs to improve IYCF practices in Bangladesh, Ethiopia, and Vietnam (19). In Bangladesh and Vietnam, large-scale SBCC interventions were implemented in various districts or provinces throughout the country from 2010 to 2014. In Ethiopia, SBCC interventions were implemented in the northwest zones of Amhara region from 2015 to 2017. A core component of all the programs was IPC (individual or group counseling or provision of key program messages). Additional interventions included MM and CM in all countries, and AG activities in Ethiopia. Descriptions of the interventions (20), evaluation design (19), and main impacts on IYCF practices in Bangladesh and Vietnam (15–17) and in Ethiopia (18) have been published previously.

In Bangladesh, BRAC, a large nongovernmental organization, delivered IPC and CM activities in 50 rural subdistricts through its existing countrywide essential health care program. In the intervention areas, BRAC health workers and community volunteers conducted multiple, age-targeted, IYCF-focused counseling visits to households with pregnant women and mothers of children ≤2 y of age (12–27 contacts, depending on frontline worker type). IYCF promotors were also recruited and trained to support them. CM included sensitization of community leaders about IYCF, and community theater shows focused on IYCF. The MM component consisted of the national broadcast of 7 television spots with messages on various aspects of IYCF—2 spots focused on breastfeeding, 4 spots focused on complementary feeding, and 1 spot focused on hygiene. In intervention areas that had low electricity and limited access to television, media-dark strategies such as local video screenings were carried out.

In Ethiopia, Alive & Thrive with Save the Children worked with government health extension workers (HEWs), health development team leaders (HDTLs; a cadre of community health volunteers), and agricultural extension workers to deliver intensified IPC about IYCF and promote AG activities to benefit children <2 y of age in 10 *woredas* (districts) in northwest Amhara. In intervention areas, HEWs provided age-appropriate IYCF messaging to women from their last trimester of pregnancy to 2 y of child age (8 contacts, 3 contacts before 6 mo of age and 5 contacts between 6 and 18 mo) during health post (i.e., first-level facility for the provision of primary healthcare for the community) and home visits and conducted food demonstrations; HDTLs also provided the age-appropriate IYCF messaging during home visits, and agricultural extension workers promoted AG activities, such as designating a chicken whose eggs are prioritized for young children in the household and designating vegetables from homestead gardens to be prioritized for children. Priests and religious leaders delivered CM activities such as sermons about adequate child feeding during religious fasting periods, which are common and extensive in the region; and enhanced community conversations about IYCF were led by community-based organizations. The MM component consisted of a regional broadcast of a radio drama, which included 12 episodes with stories that aligned with Alive & Thrive's IYCF messages, associated jingles, and testimonials of model mothers. In intervention areas with limited access to radio, there were media-dark strategies such as broadcasting the radio drama through mobile vans with speakers and traveling performers to enact parts of the drama.

In Vietnam, Alive & Thrive with Save the Children worked with the government to establish a total of 781 social franchises within government health facilities at the province, district, and commune levels in 15 of the 63 provinces to deliver high-quality IYCF counseling. Facilities were required to meet minimum criteria, including a standardized room dedicated to counseling, trained health staff (nurse midwives and doctors), and availability of job aids and client materials. The IPC schedule included 9–15 counseling contacts (≤8 breastfeeding contacts before 6 mo of age and 7 complementary feeding contacts between 6 and 24 mo) to each mother–child pair from the last trimester of pregnancy through the child's first 2 y of life. There was little to no CM, involving only the distribution of invitation cards to social franchises by village health workers to encourage mothers to attend counseling services. MM consisted of a nationally broadcast campaign that used television, print, and digital media; 2 television spots focused on breastfeeding, 1 spot focused on complementary feeding, and 1 spot promoted the use of franchise services. Other MM activities in intervention areas included community airing of loudspeaker announcements, posters promoting breastfeeding in commune health centers, billboards, and bus wraps.

### Study design and participants

The impact evaluations in all 3 countries used cluster-randomized designs. There were 2 program arms: *1*) intervention areas that received intensified IPC, CM, and MM (and AG activities in Ethiopia only); and *2*) comparison areas that received standard IPC and less-intensive CM and MM (and standard agricultural extension services in Ethiopia). Household data were collected through 2 cross-sectional surveys in the same communities, in 2010 and exactly 4 y later in 2014 (for Bangladesh and Vietnam), and in 2015 and exactly 2 y later in 2017 (for Ethiopia). Given the focus of this article on exposure to interventions, we used the endline survey data for the intervention samples only. The sample sizes included 1001 mothers with children <2 y of age in Bangladesh, 1760 mothers with children in Ethiopia (400 mothers with children aged 0–5.9 mo and 1360 mothers with children aged 6–23.9 mo), and 1001 mothers with children aged <2 y in Vietnam. Data were collected via face-to-face interviews using structured questionnaires. Informed consent was obtained from all mothers prior to their participation in the survey. Ethical approval was obtained from the institutional review boards of the Medical Research Council in Bangladesh, Addis Continental Institute of Public Health in Ethiopia, Union of Science and Technology Association in Vietnam, and the International Food Policy Research Institute in the United States. This trial was registered at www.clinicaltrials.gov as NCT01678716 (Bangladesh), NCT02775552 (Ethiopia), and NCT01676623 (Vietnam).

### Measures

#### Exposure variables

Exposure variables were based on self-reported exposure to IPC, AG, CM, and/or MM. For exposure to IPC, mothers were asked if they had been visited by a health or community worker in the last 6 mo (Bangladesh and Vietnam) or in the last 3 mo (Ethiopia) and received IYCF information. In Ethiopia only, mothers were also asked whether they heard about raising a baby's chicken or growing a baby's garden, which are AG activities with complementary feeding–related messages promoted by agricultural extension workers. For exposure to CM (in Bangladesh and Ethiopia), mothers were asked about whether they attended any of the activities related to IYCF—local theater, mobile television show, food demonstration, community conversation, and priest sermons. Exposure to MM considered ever-exposure to television spots about IYCF (Bangladesh and Vietnam) and radio drama (Ethiopia). Respondents were categorized into 4 exposure groups: *1*) any AG, CM, or MM (no IPC); *2*) IPC alone; *3*) IPC and any AG, CM, or MM; and *4*) no IPC, AG, CM, or MM.

For frequency of IPC, mothers were asked about the number of times they had IPC contacts in the last 6 mo (Bangladesh and Vietnam) or in the last 3 mo (Ethiopia). The proportions of respondents were shown by the number of contacts or 3–4 categories of contacts, depending on the distribution of respondents by frequency.

#### Outcome variables

Four IYCF practice indicators examined were as follows: *1*) exclusive breastfeeding (EBF) to 6 mo, defined as the proportion of infants 0–5 mo of age who are fed exclusively with breast milk (i.e., no foods or liquids with the exception of medications such as drops or syrups); *2*) minimum meal frequency, defined as the proportion of breastfed and nonbreastfed children 6–23 mo of age who receive solid, semisolid, or soft foods the minimum number of times or more (2 times for breastfed infants aged 6–8 mo, 3 times for breastfed children aged 9–23 mo, or 4 times for nonbreastfed children aged 6–23 mo); *3*) minimum dietary diversity, defined as the proportion of children 6–23 mo of age who receive foods from ≥4 food groups (out of 7 total food groups); and *4*) consumption of iron-rich or iron-fortified foods, defined as the proportion of children 6–23 mo of age who receive an iron-rich or iron-fortified food that is specially designed for infants and young children or that is fortified in the home (21). All IYCF indicators were constructed based on mothers’ previous-day recall about breastfeeding and of specific foods and liquids consumed by their children.

### Data analysis

Descriptive analysis was used to examine the study sample characteristics, exposure to individual or combinations of intervention components (IPC, AG, CM, and MM), and frequency of IPC contacts. Multinomial logistic regression analyses were used to test the association between the various household, maternal, and child factors and exposure groups or categories of IPC contacts. Household variables such as the number of children <5 y of age, socioeconomic status (SES), and food insecurity; maternal variables such as age, years of schooling, and ≥4 antenatal care (ANC) visits (proxy for health service–seeking behavior); and child variables such as sex, age, and illness symptoms in the last 2 wk were selected as factors that may influence intervention exposure or IYCF practice outcomes, based on a resources for care conceptual framework (22). Multivariable regression analyses were used to test the relation between the exposure to intervention components or number of IPC contacts and IYCF practices, adjusted for child age and sex and geographic clustering. Significance level was defined as *P* values <0.05. All statistical analyses were done using Stata version 15 software (StataCorp).

## Results

### Sample characteristics

Across the 3 countries, the mean number of household members was ∼5, and the number of children <5 y of age within households was similar (**Table 1**). Although all households in Ethiopia reported owning agricultural land, the proportion of food-insecure households was highest in Ethiopia, as compared with Bangladesh and Vietnam. Mothers in Bangladesh were younger than those in Ethiopia and Vietnam. Mothers in Ethiopia had the lowest mean years of schooling, followed by those in Bangladesh and Vietnam. More mothers in Bangladesh self-reported being a housewife (i.e., not employed) than those in Ethiopia and Vietnam. Maternal BMI (nutritional status) was mostly within the normal range and similar in the 3 countries, but Ethiopian mothers had the lowest dietary diversity, consuming ∼3 food groups, on average, compared to 5 and 6 food groups in Bangladesh and Vietnam, respectively. The mean age of children was higher in Ethiopia than in the other 2 countries due to a different sampling approach. The proportion of children with low birth weight was highest in Ethiopia, followed by Bangladesh and Vietnam, and reports of illness symptoms in the last 2 wk were also highest in Ethiopia. The IYCF practices among children 0–23.9 mo of age in the 3 countries at endline are presented in **Supplemental Figure 1**.

**TABLE 1:** Study sample characteristics by country^1^

Characteristics	Bangladesh (*n* = 1001)	Ethiopia (*n* = 1760)	Vietnam (*n* = 1001)
Household factors			
Household members, *n*	5.4 ± 2.1	4.9 ± 1.7	4.9 ± 1.4
Children aged <5 y, *n*	1.3 ± 0.5	1.2 ± 0.5	1.4 ± 0.6
SES index, *n*	−0.1 ± 0.9	0.0 ± 0.8	0.0 ± 0.9
Ownership of house, %	96.2	82.1	81.8
Ownership of agricultural land, %	51.6	99.9	71.1
Ownership of garden, %	31.0	17.1	72.2
Food insecurity, %	14.2	41.4	20.9
Maternal factors			
Age, y	25.3 ± 5.4	28.4 ± 6.1	28.2 ± 5.3
Schooling, y	5.9 ± 3.4	2.3 ± 3.6	10.3 ± 3.3
Housewife, %	76.2	25.3	58.7
Maternal dietary diversity, *n*	4.9 ± 1.4	2.8 ± 1.0	5.9 ± 1.5
BMI, kg/m^2^	20.9 ± 3.2	20.3 ± 2.4	20.8
≥4 ANC visits, %	57.7	58.3	78.2
Child factors			
Male, %	48.3	49.5	52.1
Age,^2^ mo	9.0 ± 6.7	11.2 ± 6.5	9.3 ± 7.1
Low birth weight, %	15.0	24.7	4.3
Underweight, %	26.1	13.8	6.0
ARI symptom in the last 2 wk, %	32.4	42.7	28.3
Diarrhea symptoms in the last 2 wk, %	3.6	20.3	4.9

1 Values are means ± SDs or percentages. ANC, antenatal care; ARI, acute respiratory infection; BMI, body mass index; SES, socioeconomic status.

2 In Ethiopia, over two-thirds of the households had children aged 6–23.9 mo; thus, mean child age is higher than in the other 2 countries.

### Intervention coverage and factors associated with exposure

Exposure to IPC about IYCF, with or without the other intervention components, was highest in Bangladesh at 81%, followed by 49% in Ethiopia, and 40% in Vietnam (**Table 2**). Additional detailed indicators on IPC exposure are presented in **Supplemental Table 1**. Coverage of the AG activities in Ethiopia was 35%. CM coverage was 33% in Bangladesh and 46% in Ethiopia. Coverage of MM, specifically television spots, was >70% in Bangladesh and Vietnam, whereas coverage of the radio drama in Ethiopia was 34%. Overall, only 5%, 22%, and 16% of mothers in the intervention areas were not exposed to any of the intervention components in Bangladesh, Ethiopia, and Vietnam, respectively.

**TABLE 2:** Exposure to interventions by country^1^

Indicator	Bangladesh (*n* = 1001)	Ethiopia (*n* = 1760)	Vietnam (*n* = 1001)
IPC			
Visited in the last 6/3 mo and received IYCF advice^2^	80.92	48.64	39.68
AG			
Heard about baby's chicken	—	33.58	—
Heard about baby's garden	—	20.74	—
Any AG (above)	—	35.1	—
CM			
Seen theater	13.79	—	—
Seen television show	29.27	—	—
Attended food demonstration	—	33.75	—
Attended community conversation	—	12.67	—
Heard priest sermons	—	20.11	—
Any CM (above)	33.27	45.5	
MM			
Ever heard radio drama	—	33.75	—
Ever seen television spot IYCF	73.63	—	76.79
None/no intervention exposure	5.19	21.93	16.17
Combinations of intervention components			
IPC alone	19.18	8.18	7.04
AG alone	—	4.26	—
CM alone	0.40	7.27	—
MM alone	9.09	6.59	44.15
No IPC + any AG/CM/MM	13.89	29.43	44.15
IPC + any AG/CM/MM	61.74	40.45	32.64

1 Values are percentages. AG, (nutrition-sensitive) agriculture; CM, community mobilization; IPC, interpersonal communication; IYCF, infant and young child feeding practice; MM, mass media.

2 Recall periods are 6 mo in Bangladesh and Vietnam and 3 mo in Ethiopia.

There was substantial overlap in exposure to IPC and other intervention components—that is, mothers received multiple intervention components (IPC, AG, CM, and/or MM) (Table 2). In Bangladesh, 19% of mothers were exposed to IPC alone, 14% were exposed to MM or CM without IPC, and 62% were exposed to IPC with other intervention components. In Ethiopia, 8%, 29%, and 40% of mothers were exposed to IPC alone, other intervention components without IPC, and IPC with other intervention components, respectively. In Vietnam, 7%, 44%, and 33% of mothers were exposed to IPC alone, MM alone, and IPC with MM, respectively. Thus, in Bangladesh and Ethiopia, most mothers were exposed to IPC along with other intervention components; in Vietnam, most mothers were exposed to MM alone.

Patterns of association between household, maternal, and child factors and intervention exposure differed across the 3 countries (**Table 3**). In Bangladesh, mothers with higher SES were more likely to be exposed to CM or MM, with or without IPC. Mothers with older children or children with low birth weight were more likely to be exposed to CM or MM without IPC, while older mothers were less likely to be in this exposure group. Mothers with higher BMI (but still within the normal range, which may reflect higher SES) were more likely to be exposed to CM or MM without IPC or IPC alone, and mothers who received ≥4 ANC visits were more likely to be in any exposure group. In Ethiopia, mothers with higher SES were more likely to be exposed to AG, CM, or MM, with or without IPC, much like in Bangladesh. Mothers with higher schooling and children with low birth weight were more likely to be exposed to AG, CM, or MM without IPC; and those with older children were less likely to be exposed to IPC alone. Mothers with higher food insecurity, older age, and those who received ≥4 ANC visits were more likely to be exposed to IPC with any AG, CM, or MM, while those with children who experienced acute respiratory infection symptoms in the last 2 wk were less likely. In Vietnam, mothers with higher SES and higher schooling were more likely to be exposed to IPC and MM. Older mothers were less likely to be exposed to MM without IPC. Those who reported being a housewife were less likely to be exposed to IPC, with or without MM. Mothers with higher BMI and older children were more likely to be exposed to MM without IPC, and those with older children were less likely to be exposed to IPC alone.

**TABLE 3:** Factors associated with exposure to intervention components by country^1^

	Bangladesh			Ethiopia			Vietnam		
Characteristic	No IPC + any CM/MM (*n* = 139)	IPC alone (*n* = 192)	IPC + any CM/MM (*n* = 618)	No IPC + any AG/CM/MM (*n* = 518)	IPC alone (*n* = 144)	IPC + any AG/CM/MM (*n* = 712)	No IPC + MM (*n* = 445)	IPC alone (*n* = 71)	IPC + MM (*n* = 329)
Household factors									
No. of household members	1.11	1.00	1.00	1.09	1.15	1.09	1.04	1.03	0.95
No. of children <5 y	1.31	1.18	1.83	0.74	0.67	0.81	1.08	0.67	1.07
SES index	1.62*	0.93	1.54*	1.46***	1.08	1.33**	1.16	1.50	1.37*
Food insecurity	1.71	1.74	1.27	1.12	1.27	1.37*	0.86	1.05	0.74
Maternal factors									
Age	0.89***	0.95	0.95	1.03	1.04	1.04*	0.95**	1.01	0.93***
Schooling	0.97	0.94	0.97	1.05*	1.06	1.03	0.99	1.06	1.08*
Housewife	0.78	0.61	0.68	0.89	0.85	0.80	1.16	0.42*	0.60*
BMI	1.16*	1.12*	1.07	0.96	0.98	0.96	1.09*	0.96	1.04
≥4 ANC visits	2.27*	2.96**	2.55**	1.24	1.16	1.77***	1.32	1.04	1.01
Child factors									
Male	1.24	1.21	1.34	1.11	1.23	1.26	1.00	0.85	0.85
Age	1.06*	1.01	1.04	1.01	0.96*	1.02	1.04**	0.94*	1.02
Low birth weight	2.70*	1.04	1.58	1.46*	1.22	0.84	0.86	0.22	0.57
Underweight	0.59	0.54	0.58	0.89	1.00	0.97	0.70	0.87	0.60
ARI symptom in the last 2 wk	0.85	0.58	0.90	0.88	0.91	0.73*	1.49	0.65	0.99
Diarrhea symptoms in the last 2 wk	0.79	1.08	0.85	1.35	1.15	1.23	0.83	0.60	1.47

1 Values are ORs, with no exposure as the reference group. *P < 0.05, **P < 0.01, ***P < 0.001. AG, (nutrition-sensitive) agriculture; ANC, antenatal care; ARI, acute respiratory infection; BMI, body mass index; CM, community mobilization; IPC, interpersonal communication; MM, mass media; SES, socioeconomic status.

### Frequency of IPC contacts and factors associated with number of contacts

In relation to the frequency of IPC contacts, mothers with children 0–5.9 mo of age reported an average of 6.6 IPC visits in the last 6 mo in Bangladesh, 1.8 visits in the last 3 mo in Ethiopia, and 0.9 visits in the last 6 mo in Vietnam (**Table 4**). Among mothers with children aged 6–23.9 mo, an average of 9.0, 2.3, and 0.8 visits were reported in Bangladesh, Ethiopia, and Vietnam, respectively.

**TABLE 4:** Number of IPC contacts by child age group and country^1^

No. of visits in the last 6/3 mo^2^	Bangladesh^3^	Ethiopia	Vietnam
Age 0–5.9 mo, *n*	501	400	501
0	7.58	21.50	58.28
1	5.99	19.50	17.96
2	8.38	18.50	12.38
3	4.19	40.50	5.39
4	11.98	—	3.59
5	5.19	—	2.40
6	9.58	—	0
>6	47.11	—	—
Mean no. of visits	6.59 ± 4.28	1.78 ± 1.19	0.86 ± 1.31
Age 6–23.9 mo, *n*	500	1360	500
0	4.40	23.16	60.60
1	2.00	23.24	18.60
2	5.40	17.72	11.00
3	2.60	35.88	4.40
4	4.40	—	2.80
5	2.60	—	2.60
6	8.60	—	0
>6	70.40	—	—
Mean no. of visits	9.04 ± 4.24	2.27 ± 2.28	0.83 ± 1.58
Age 0–23.9 mo, *n*	1001	1760	1001
0	5.99	22.78	59.44
1	4.00	22.39	18.28
2	6.89	17.90	11.69
3	3.40	36.93	4.9
4	7.99	—	3.2
5	3.90	—	2.5
6	9.09	—	0
>6	58.74	—	—
Mean no. of visits	7.81 ± 4.43	2.33 ± 2.35	0.85 ± 1.45

1 Values are percentages or means ± SDs unless otherwise indicated. IPC, interpersonal communication; IYCF, infant and young child feeding practice.

2 Recall periods are 6 mo in Bangladesh and Vietnam and 3 mo in Ethiopia.

3 Visits by community health volunteer and/or IYCF promoter.

Patterns of association between the household, maternal, and child factors and categories of IPC contacts differed across countries (**Table 5**). In Bangladesh, mothers with higher numbers of household members were less likely to achieve >6 IPC contacts. Those who received ≥4 ANC visits or with older children were more likely to achieve >6 contacts. Older mothers were more likely to achieve 1–3 or >6 contacts. In Ethiopia, mothers with higher food insecurity or who received ≥4 ANC visits were more likely to achieve ≥3 contacts, while those with older children were less likely to achieve this. Older mothers and those with higher schooling were more likely to achieve any number of IPC contacts, but mothers who reported being a housewife were less likely to achieve any number of contacts. In Vietnam, mothers with higher SES were more likely to achieve 1 IPC visit, and those with higher schooling were more likely to achieve ≥2 visits. Mothers who reported being a housewife were less likely to achieve any number of visits, and those with children with low birth weight were less likely to achieve ≥2 visits.

**TABLE 5:** Factors associated with numbers of IPC contacts by country^1^

	Bangladesh			Ethiopia		Vietnam	
Characteristic	1–3 visits (*n* = 143)	4–6 visits (*n* = 210)	>6 visits (*n* = 588)	1–2 visits (*n* = 709)	≥3 visits (*n* = 650)	1 visit (*n* = 183)	≥2 visits (*n* = 223)
Household factors							
No. of household members	0.87	0.88	0.82**	0.99	1.00	0.90	0.97
No. of children <5 y	1.74	1.30	1.71	1.02	1.14	0.93	0.93
SES index	1.14	1.11	0.90	1.05	1.05	1.39**	1.11
Food insecurity	0.85	0.45	0.57	1.27	1.37*	0.84	0.94
Maternal factors							
Age	1.07*	1.05	1.06*	1.04*	1.05***	0.99	0.98
Schooling	0.99	0.93	0.91	1.06**	1.06**	1.06	1.12***
Housewife	0.70	0.56	0.51	0.61***	0.55***	0.48***	0.48***
BMI	0.95	0.93	0.95	0.97	1.01	0.96	0.97
≥4 ANC visits	1.04	1.25	2.24**	1.07	1.34*	0.69	0.90
Child factors							
Male	0.76	0.83	1.00	0.79	1.12	0.75	0.90
Age	0.97	0.98	1.06**	0.98	0.98*	0.98	0.96**
Low birth weight	0.87	1.14	1.06	0.81	0.88	0.66	0.59
Underweight	1.10	1.03	0.95	0.95	0.85	0.67	0.78
ARI symptom in the last 2 wk	0.86	0.72	0.85	1.09	1.24	0.74	0.72
Diarrhea symptoms in the last 2 wk	0.99	0.93	0.50	1.06	0.85	1.29	1.74

1 Values are ORs, with no visits as the reference group. *P < 0.05, **P < 0.01, ***P < 0.001. ANC, antenatal care; ARI, acute respiratory infection; BMI, body mass index; IPC, interpersonal communication; SES, socioeconomic status.

### Association between intervention exposure and IYCF practices

In Bangladesh, none of the exposure groups was associated with IYCF practices, as compared with no exposure, which was very low (**Table 6**). In Ethiopia, mothers exposed to AG, CM, or MM without IPC were 0.5 times less likely to practice EBF. IPC alone was associated with 1.7 higher odds of minimum meal frequency, however, and IPC combined with any other intervention component was associated with higher odds of minimum meal frequency (OR: 1.6), minimum dietary diversity (OR: 1.8), and consumption of iron-rich foods (OR: 4.7). In Vietnam, mothers exposed to IPC alone or IPC with MM were 3.7 and 2.8 times more likely to practice EBF, respectively. IPC alone was associated with 0.5 lower odds of consumption of iron-rich foods, but the prevalence of iron-rich food consumption was already very high at >97%.

**TABLE 6:** Association between intervention exposure and IYCF practices among mothers with children aged 0–23.9 mo^1^

Indicator	Exclusive breastfeeding: age 0–5 mo	Minimum meal frequency: age 6–23 mo	Minimum dietary diversity: age 6–23 mo	Consumption of iron-rich food: age 6–23 mo
Bangladesh				
Ref: None	1.00	1.00	1.00	1.00
No IPC + any CM or MM	2.52	1.54	2.19	1.46
IPC alone	2.83	1.10	1.43	0.77
IPC + any CM or MM	1.23	1.75	1.92	1.16
Ethiopia				
Ref: None	1.00	1.00	1.00	1.00
No IPC + any AG, CM, or MM	0.46**	0.99	0.79	2.33
IPC alone	0.54	1.72*	1.00	2.41
IPC + any AG, CM, or MM	1.00	1.61*	1.75***	4.70***
Vietnam				
Ref: None	1.00	1.00	1.00	1.00
No IPC + MM	1.56	2.49	1.48	0.45
IPC alone	3.70**	1.38	1.05	0.50**
IPC + MM	2.82**	3.81	3.26	0.39

1 Values are ORs. *P < 0.05, **P < 0.01, ***P < 0.001; adjusted for child age, sex, geographic clustering, and variables that were significantly different between exposure groups (socioeconomic status, food security, maternal age, BMI, ≥4 antenatal care visits, acute respiratory infection, and diarrhea symptoms). AG, (nutrition-sensitive) agriculture; CM, community mobilization; IPC, interpersonal communication; IYCF, infant and young child feeding practice; MM, mass media; Ref, reference.

### Association between number of IPC contacts and IYCF practices

In Bangladesh, mothers who received 4–6 visits in the last 6 mo were 3.4 times more likely to practice EBF; those who received 1–3 visits and >6 visits were 2.5 and 3.4 times more likely to achieve minimum meal frequency, respectively (**Table 7**). In Ethiopia, 1–2 visits and ≥3 visits in the last 3 mo were associated with 1.5 and 2.8 times higher odds of minimum dietary diversity, respectively. Three or more visits were also associated with 3.0 higher odds of consumption of iron-rich foods. In Vietnam, any frequency of visits was associated with ≥2 times higher odds of EBF.

**TABLE 7:** Association between the frequency of contacts and IYCF practices among mothers with children aged 0–23.9 mo^1^

Indicator	Exclusive breastfeeding: age 0–5 mo	Minimum meal frequency: age 6–23 mo	Minimum dietary diversity: age 6–23 mo	Consumption of iron-rich food: age 6–23 mo
Bangladesh				
No. of visits in the last 6 mo				
Ref: 0	1.00	1.00	1.00	1.00
1–3	1.22	2.53*	1.14	0.94
4–6	3.44*	2.05	0.53	0.77
>6	2.14	3.38**	0.69	0.82
Ethiopia				
No. of visits in the last 3 mo				
Ref: 0	1.00	1.00	1.00	1.00
1–2	1.44	1.19	1.46**	1.24
≥3	1.08	1.22	2.76***	3.04*
Vietnam				
No. of visits in the last 6 mo				
Ref: 0	1.00	1.00	1.00	1.00
1	2.11*	2.48	1.88	0.79
≥2	2.18**	3.24	2.94	0.52

1 Values are ORs. *P < 0.05, **P < 0.01, ***P < 0.001; adjusted for child age, sex, geographic clustering, and variables that were significantly different between frequency groups (socioeconomic status, food security, maternal age, BMI, ≥4 antenatal care visits, acute respiratory infection, and diarrhea symptoms). IYCF, infant and young child feeding practice; Ref, reference.

## Discussion

Understanding the extent of intervention exposure and the number of contacts achieved, as well as who are reached by large-scale behavior change interventions, and their association with outcomes, provides important insights on what can be implemented feasibly at scale, what intensities matter, and the context of implementation. In all 3 countries, we observed large overlap in exposure to IPC with other intervention components; a higher proportion of women were exposed to other interventions (i.e., MM) without IPC than with IPC in Vietnam only. On average, mothers received 8 visits in the last 6 mo, 2 visits in the last 3 mo, and 1 visit in the last 6 mo in Bangladesh, Ethiopia, and Vietnam, respectively. Our study results reflect different contexts in the 3 countries as seen by the different patterns of factors associated with intervention exposure and number of IPC contacts. In Bangladesh, there were positive, nonsignificant associations between any intervention exposure and IYCF practices, but we observed significant associations with ≥4 contacts. In Ethiopia, exposure to IPC with other interventions was associated with higher odds of minimum meal frequency, minimum dietary diversity, and consumption of iron-rich foods. In Vietnam, exposure to IPC alone or with MM was associated with higher odds of EBF. Near-monthly or more frequent visits were associated with higher odds of IYCF practices in Bangladesh and Ethiopia. In Vietnam, even 1 IPC visit was associated with higher odds of EBF, but most mothers who had an IPC visit were also exposed to MM.

In the intervention areas, large overlap of IPC with other intervention components (CM, MM, and/or AG) was observed in Bangladesh and Ethiopia, where IPC was mainly community based and delivered through home visits. The other interventions extended reach to the target population not covered by IPC, but mostly they overlapped with and likely played a role in reinforcing the IPC contacts. In Vietnam, IPC contacts were facility-based only, and therefore more mothers were exposed to MM than IPC. Overall, 77% of mothers were exposed to any MM and 44% of them were exposed to MM alone; thus, MM played an important role in extending the reach of behavior change communication or IYCF messages to mothers in Vietnam, which was also documented during the process evaluation (23).

The expected frequency of IPC contacts according to intervention design was mostly achieved in Bangladesh and Ethiopia but fell short in Vietnam. By design, Bangladeshi mothers were expected to receive at least monthly contacts (12–27 total contacts for children ≤2 y of age), Ethiopian mothers were to receive bimonthly contacts (3 contacts between 0 and 5 mo of age and 5 contacts after 6 mo; 8 total contacts), and Vietnamese mothers were to receive 8 contacts before 6 mo of age and 7 contacts between 6 and 23 mo (9–15 total contacts). In Bangladesh, extensive cadres of BRAC frontline workers, dedicated to delivering health- and nutrition-promotion activities through home visits, were able to deliver monthly or more frequent IPC contacts through home visits. In Ethiopia, where government HEWs and community health volunteers delivered IPC through health post or home visits, respectively, ∼2 contacts over the 3-mo recall period were reported by mothers. In Vietnam, however, <1 contact over a 6-mo recall period was reported by mothers. Facility-based IPC delivered through government health centers was not able to achieve the planned regimen, reflecting the added challenge of generating a demand for these services so that mothers will go to the health facilities and attend them, compared with services delivered directly to their homes. Other constraints to utilization of the facility-based interventions included the distance to facilities and the lack of perceived need (the perception of need for IYCF counseling was less recognized than it is for illness) (24). Inasmuch as SBCC strategies had to be tailored to the different drivers and barriers (i.e., traditional feeding habits or food taboos/myths, locally available foods, work away from home, etc.) and situation (low to high prevalence) of the promoted behaviors (25), the delivery platforms and frontline worker characteristics differed in the 3 countries, which influenced service performance and likely contributed to the different numbers of contacts achieved (26).

The patterns of household, maternal, and child factors associated with exposure to different combinations of intervention components and number of IPC contacts reflect different contexts across the 3 countries. In Bangladesh, mothers with higher SES, reflecting more household resources, were more likely to be exposed to other intervention components (CM or MM), with or without IPC. Mothers who received ≥4 ANC visits, reflecting health service use, were more likely to be in any exposure group. Those with older children and children with low birth weight were more likely to be exposed to other interventions without IPC. There was a mix of positive and negative conditions associated with exposure groups in Bangladesh, as well as in Ethiopia, reflecting a complex interplay of demand-side factors on how community workers might target beneficiaries and how beneficiaries access or use SBCC interventions. In Ethiopia, those with higher food insecurity and older maternal age were more likely to be exposed to IPC with other interventions, which may be a result of community health workers targeting poorer or more vulnerable households. Mothers who received ≥4 ANC visits, reflecting health service–seeking behavior, were also more likely exposed to IPC with other interventions.

In Vietnam, mothers with higher SES and higher schooling were more likely exposed to IPC and MM, reflecting associations with positive resources and maternal capacity. Older mothers were less likely to be exposed to MM, with or without IPC. Mothers who reported being a housewife were less likely to be exposed to IPC alone or IPC with MM, indicating that they did not use or used less often the facility-based IPC than mothers who reported being employed, which appears to run counter to the idea that housewives may have more time to access IPC services. Mothers with older children were more likely to be exposed to MM alone and less likely to be exposed to IPC alone. A closer examination of factors associated with one-time, repeated, or recommended use of the facility-based IPC services in Vietnam was presented in a separate article (24). Overall, the different contexts of implementation of interventions across the countries reflected the different means of intervention delivery as well as the characteristics of the target population.

In relation to the number of IPC contacts, older maternal age, older child age, and receipt of ≥4 ANC visits were associated with more IPC contacts in Bangladesh, while those with more household members were less likely to receive >6 visits. Since IPC in Bangladesh was entirely home based, these relationships may reflect a combination of factors related to service demand (service-seeking) and delivery challenge (with more people residing in the house). In Ethiopia, higher food insecurity and receipt of ≥4 ANC visits were associated with ≥3 IPC visits, either through health post or home visits. Older age and more schooling among mothers were associated with any number of IPC contacts. In both Ethiopia and Vietnam, being a housewife and older child age were associated with fewer IPC visits, suggesting that housework and obligations at the home may occupy as much or more of women's time compared to employment (24).

The associations between combinations of intervention exposure or number of IPC contacts and IYCF practices present different scenarios across countries. In Bangladesh, any exposure appeared to be insufficient, and the frequency of contacts mattered for IYCF practices. Also, higher exposure to IPC was not associated with minimum dietary diversity or consumption of iron-rich foods in Bangladesh, which may suggest that these practices require inputs such as a variety of or specific nutrient-rich foods and are not adequately influenced by behavior change communication alone in this population. In Ethiopia, the combination of exposure to IPC and other intervention components and at least monthly IPC contacts was important for complementary feeding practices. In Vietnam, exposure to IPC alone and IPC with MM was associated with higher odds of EBF, as was exposure to ≥1 IPC contact. Even just 1 facility-based IPC contact appeared to be effective for EBF in Vietnam, which is corroborated by findings of significant improvements in IYCF counseling service quality as a result of incorporating the social franchising elements into government health care facilities (27).

Our findings contribute to the evidence base on the implementation of behavior change interventions, particularly the combination of interventions and frequency of IPC or counseling contacts to improve child feeding practices. A systematic review on breastfeeding counseling showed that ≥4 contacts may reduce the likelihood of not exclusively breastfeeding, compared with standard care or no breastfeeding counseling (8). Our study used data on large-scale intervention program evaluations in 3 country contexts to examine both breastfeeding and complementary feeding practices. The context of delivery (e.g., home visits by community health workers or facility-based counseling sessions) and of the target population (e.g., household SES, maternal schooling, child age) influenced intervention uptake and benefits.

Data from cross-sectional household surveys were used to assess intervention exposure, factors associated with exposure, and IYCF practices; thus, we cannot determine causal relations between them. The impact evaluation results have already proven the effectiveness of interventions, however, and our present findings are the result of closer examination at how these interventions were implemented and associated with the outcomes. Measures were based on maternal report and may be susceptible to recall bias. Feasible ranges and variability in indicators corroborated by process evaluation results and program reports strengthen our interpretation of results. Also, EBF practice was measured in our study based on mothers’ recall in the last 24 h rather than over the entire period since birth, but this standard point-in-time measure of current status helps to avoid the risk of recall error over a longer period and serves its purpose in our study for comparison across the 3 countries (28). While SBCC interventions and the implementation conditions in any 2 countries cannot be exactly the same, our study compared similar approaches designed and led by the same initiative (Alive & Thrive) with the same outcomes. Thus, we believe these 3 country cases warranted this comparative analysis.

In conclusion, the combination of behavior change interventions and number of IPC contacts required to achieve IYCF behavior change seem to be very context specific. Further research to understand the relations between intervention exposure, individual characteristics, contexts, and benefits can help guide decisions about intervention planning and implementation at scale.

## Supplementary Material

nzz140_Supplemental_File

## Notes

This study was funded by the Bill & Melinda Gates Foundation, through Alive & Thrive, managed by FHI 360. Additional financial support to the evaluation study was provided by the CGIAR Research Program on Agriculture for Nutrition and Health (A4NH), led by the International Food Policy Research Institute.

Author disclosures: The authors report no conflicts of interest. None of the authors received gifts or honoraria associated with this project, but travel support for the authors (to study sites and conferences) was included in the project grant.

This article is based on secondary analysis of completed impact evaluations. Primary analysis results were published separately in full collaboration with authors from each country (Bangladesh, Ethiopia, and Vietnam).

Supplemental Table 1 and Supplemental Figure 1 are available from the "Supplementary data" link in the online posting of the article and from the same link in the online table of contents at https://academic.oup.com/cdn/.

Abbreviations used: AG, (nutrition-sensitive) agriculture/agricultural; ANC, antenatal care; CM, community mobilization; EBF, exclusive breastfeeding; HDTL, health development army team leader; HEW, health extension worker; IPC, interpersonal communication; IYCF, infant and young child feeding; MM, mass media; SBCC, social and behavior change communication; SES, socioeconomic status.
