# Supplementary material for: Different Combinations of Behavior Change Interventions and Frequencies of Interpersonal Contacts Are Associated with Infant and Young Child Feeding Practices in Bangladesh, Ethiopia, and Vietnam
Source: Curr Dev Nutr. 2019 Dec 9;4(2):nzz140. doi: 10.1093/cdn/nzz140 (PMC6964730; doi:10.1093/cdn/nzz140)
Supplement: nzz140_Supplemental_File [file nzz140_supplemental_file.docx]

**Supplemental Figure 1: IYCF practices among children aged 0- 23.9 months for Bangladesh, Ethiopia and Viet Nam**

**Supplemental Table 1: Exposure to IPC contacts by country^1^**

|  | **Bangladesh** | | **Ethiopia** | | **Viet Nam** |
| --- | --- | --- | --- | --- | --- |
| **Indicator** | **SS** | **SK** | **HEW/HP** | **HDTL** | **HS** |
| % visited at home/HF in the last 6 mo | 87.41 | 29.87 |  |  | 40.56 |
| % visited at home/HF in the last 30 days | 78.32 | 16.18 |  |  |  |
| % visited at home/HF in the last 3 months |  |  | 83.41 | 42.05 |  |
| % visited at home/HF during pregnancy | 69.13 | 42.16 | 69.13 | 72.48 | 42.76 |
| No of visits (total) | 7.40 ± 6.92 | 1.25 ± 2.37 | 1.47 ± 2.15 | 1.02 ± 1.95 | 2.26 ± 3.40 |
| No of visits in the last 6 mo | 4.31 ± 2.85 | 0.97 ± 1.97 |  |  | 0.85 ± 0.05 |
| No of visits in the last 3 mo |  |  | 1.83 ± 1.78 | 0.50 ± 1.08 |  |
| No of visits in the last 30 days | 1.12 ± 0.80 | 0.18 ± 0.44 |  |  |  |
| No of visits during pregnancy | 1.22 ± 1.08 | 0.64 ± 0.85 |  |  | 1.00 ± 1.59 |
| Received BF advice in the last contact | 49.65 | 15.08 | 33.18 | 13.01 | 29.76 |
| Received CF advice in the last contract | 45.85 | 9.59 | 33.18 | 14.26 | 28.67 |
| Received IYCF advice in the last contact | 71.23 | 18.98 | 44.15 | 19.26 | 39.68 |

^1^Values are percentages or mean ±SD. BF: breastfeeding; CF: complementary feeding; HDTL: health development army team leader [community volunteer], HEW/HP: health extension worker/health post, HS: health staff, IYCF: infant and young child feeding; SK: *Shasthya Kormi* [health worker], SS: *Shasthya Sebika* [community health volunteer].
